# Bolbase: a comprehensive genomics database for *Brassica oleracea*

**DOI:** 10.1186/1471-2164-14-664

**Published:** 2013-09-30

**Authors:** Jingyin Yu, Meixia Zhao, Xiaowu Wang, Chaobo Tong, Shunmou Huang, Sadia Tehrim, Yumei Liu, Wei Hua, Shengyi Liu

**Affiliations:** 1 The Key Laboratory of Oil Crops Biology and Genetic Breeding, the Ministry of Agriculture, Oil Crops Research Institute, the Chinese Academy of Agricultural Sciences, Wuhan 430062, China; 2 Institute of Vegetables and Flowers, the Chinese Academy of Agricultural Sciences, Beijing 100081, China; 3 Department of Agronomy, Purdue University, West Lafayette 47907, IN, USA

**Keywords:** *Brassica oleracea*, Database, Genome sequence, Synteny, Comparative genomics

## Abstract

**Background:**

*Brassica oleracea* is a morphologically diverse species in the family Brassicaceae and contains a group of nutrition-rich vegetable crops, including common heading cabbage, cauliflower, broccoli, kohlrabi, kale, Brussels sprouts. This diversity along with its phylogenetic membership in a group of three diploid and three tetraploid species, and the recent availability of genome sequences within *Brassica* provide an unprecedented opportunity to study intra- and inter-species divergence and evolution in this species and its close relatives.

**Description:**

We have developed a comprehensive database, Bolbase, which provides access to the *B. oleracea* genome data and comparative genomics information. The whole genome of *B. oleracea* is available, including nine fully assembled chromosomes and 1,848 scaffolds, with 45,758 predicted genes, 13,382 transposable elements, and 3,581 non-coding RNAs. Comparative genomics information is available, including syntenic regions among *B. oleracea*, *Brassica rapa* and *Arabidopsis thaliana,* synonymous (Ks) and non-synonymous (Ka) substitution rates between orthologous gene pairs, gene families or clusters, and differences in quantity, category, and distribution of transposable elements on chromosomes. Bolbase provides useful search and data mining tools, including a keyword search, a local BLAST server, and a customized GBrowse tool, which can be used to extract annotations of genome components, identify similar sequences and visualize syntenic regions among species. Users can download all genomic data and explore comparative genomics in a highly visual setting.

**Conclusions:**

Bolbase is the first resource platform for the *B. oleracea* genome and for genomic comparisons with its relatives, and thus it will help the research community to better study the function and evolution of *Brassica* genomes as well as enhance molecular breeding research. This database will be updated regularly with new features, improvements to genome annotation, and new genomic sequences as they become available. Bolbase is freely available at http://ocri-genomics.org/bolbase.

## Background

*Brassica oleracea* (CC genome, 2n = 18) is one of the most important species in the family Brassicaceae, which also contains the model species *Arabidopsis thaliana* and a great number of nutrition-rich vegetables and oilseed crops, such as *B. rapa* (AA, 2n = 20), *B. nigra* (BB, 2n = 16), *B. napus* (AACC, 2n = 38), *B. carinata* (BBCC, 2n = 34) and *B. juncea* (AABB, 2n = 36) [1]. *Brassica oleracea* is a very morphologically diverse species that includes common heading cabbage (*B. oleracea* ssp. *capitata* L.), cauliflower (*B. oleracea* ssp. *botrytis* L.), broccoli (*B. oleracea* ssp. *italica* L.), kohlrabi (*B. oleracea* ssp. *gongylodes* L.), kale (*B. oleracea* ssp. *medullosa* Thell.), and Brussels sprouts (*B. oleracea* ssp. *gemmifera* DC) [2]. This intriguingly broad variation provides an excellent model for studying biological functionality and morphological evolution using the modern tools of molecular evolutionary biology and comparative genomics [3,4].

The *A. thaliana* genome has undergone two whole genome duplication events (α and β) within the crucifer lineage and one more ancient genome triplication event (γ) shared with most dicots (asterids and rosids) [5]. The *Brassica* and *Arabidopsis* lineages diverged from a common ancestor about 20 million years ago (MYA) after the α events [6], and a whole genome triplication event occurred subsequently in the *Brassica* ancestor 13–17 MYA [7]. The two representative *Brassica* diploids, *B. rapa* and *B. oleracea*, separated from each other about 3.75 MYA [8]. The genetic system of *Brassica* species, particularly of those described by the "triangle of U" (the relationship between three diploids and three synthetic tetraploids) [1], provides an unprecedented opportunity to study inter-species hybridization, polyploidization, genome evolution and its role in plant speciation. The genome of *B. rapa* (A genome) has been sequenced and made available in the BRAD database [9]. Recently, we finished the genome assembly of *B. oleracea* (C genome) and submitted the data to NCBI. These primary genomic data will facilitate structural, functional, and evolutionary analyses of *Brassica* genomes, as well as those of other Brassicaceae.

**Figure 1:**
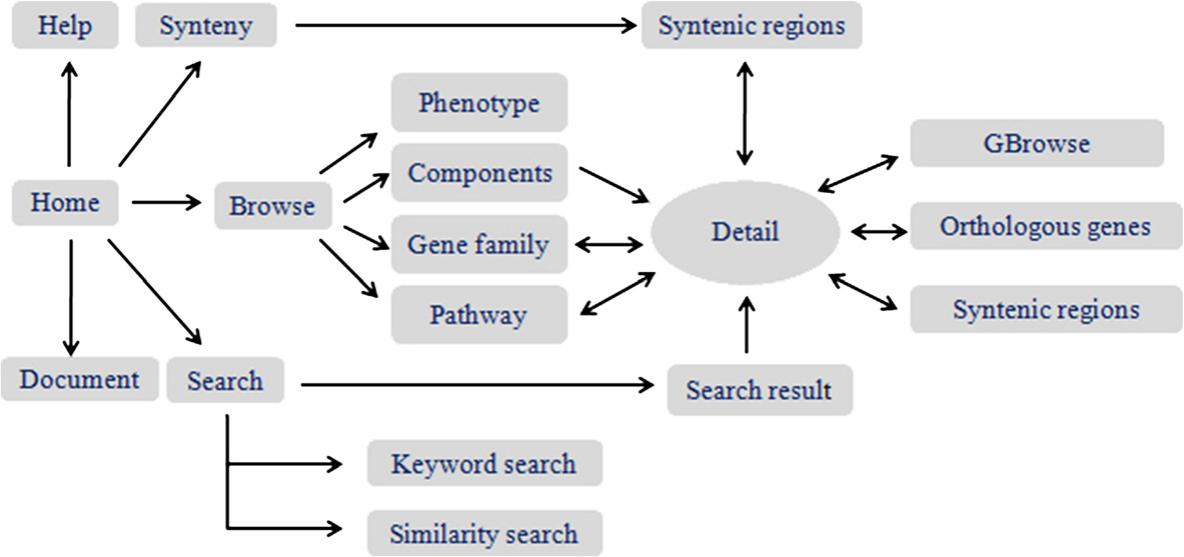
Schematic illustration of the Bolbase sitemap.

There now exist several public databases for *B. oleracea* genome sequence data, including Brassica Genome Gateway (http://brassica.bbsrc.ac.uk/), Brassica.info (http://www.brassica.info/resource/databases.php), and AAFC Comparative Genome Viewer (http://brassica.agr.gc.ca/navigation/viewer_e.shtml). These databases present only partial genomic data for *B. oleracea*, such as QTLs, ESTs and cloned genes. To better access, search, visualize, and understand the genome sequences, annotation, structure, and evolution of the *B. oleracea* genome, we developed a comprehensive web-based database, Bolbase (http://ocri-genomics.org/bolbase), which include genome sequence data and comparative genomics information. This user-friendly database will serve as an infrastructure for researchers to study the molecular function of genes, comparative genomics, and evolution in closely related Brassicaceae species as well as promote advances in molecular breeding within *Brassica* (Figure 1).

## Construction and content

The genome of *B. oleracea capitata* (line 02–12) was sequenced by next generation sequencing technologies combined with 454 and Sanger sequencing. In total, a 540-Mb draft assembly, representing 85% of the estimated 630-Mb genome, was generated and submitted to NCBI. In Bolbase, we collected the complete sequence assembly, including nine pseudomolecular chromosomes, 1,848 scaffolds, and all genome components, comprising 45,758 predicted protein-coding genes, 13,382 transposable elements, and 3,581 non-coding RNAs. For each annotated genomic component, we supplied detailed annotations and cross-links to publicly available databases. Moreover, we provided a comprehensive analysis of synteny among *B. oleracea*, *B. rapa*, and *A. thaliana* using data from BRAD (http://brassicadb.org/brad/, v1.0) [9] and TAIR (http://www.arabidopsis.org, TAIR9) [10], respectively.

### Genomic component

**Figure 2:**
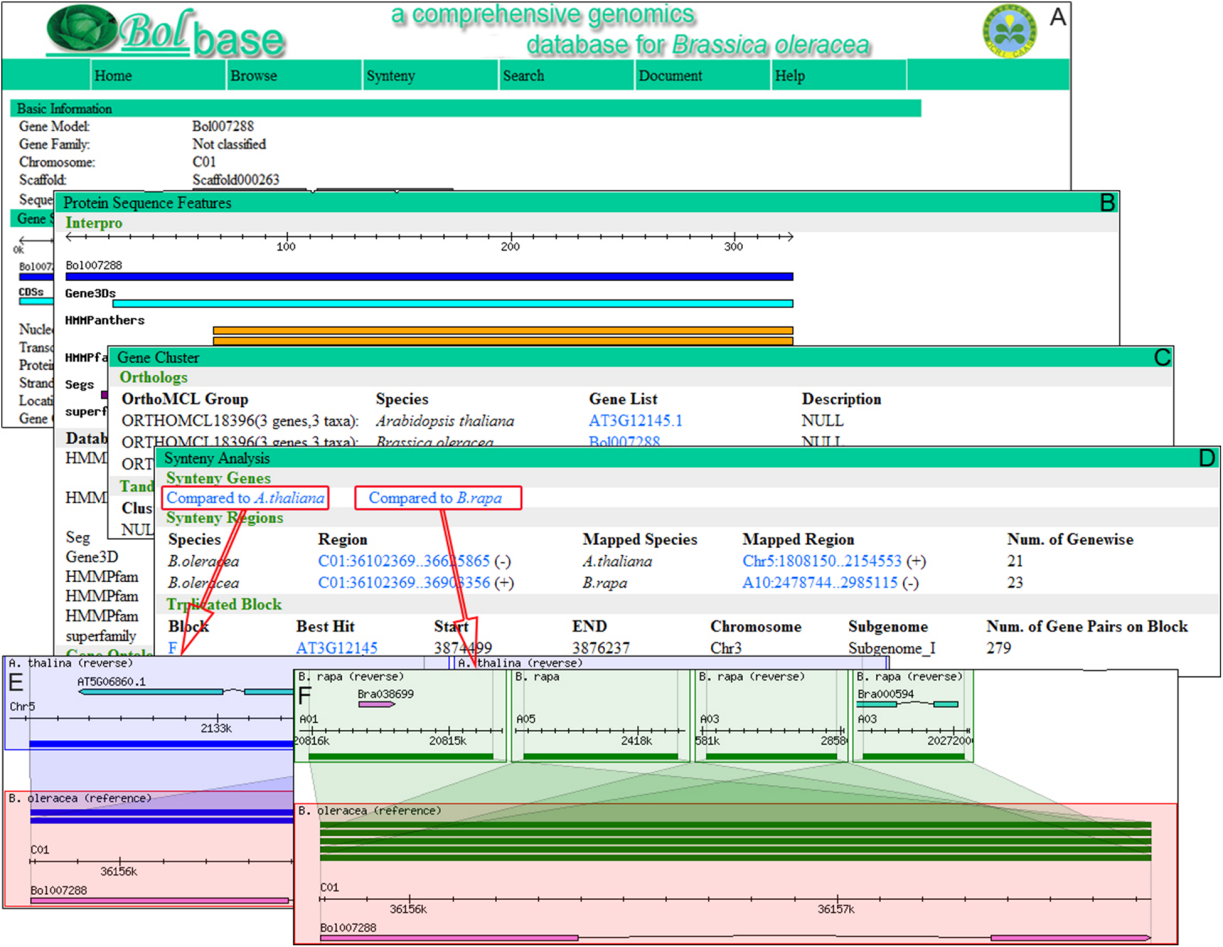
**Annotation of predicted protein-coding genes in the *Brassica oleracea *genome. A**. basic information; **B**. protein sequence features; **C**. gene clusters, including orthologous groups and tandem duplicated arrays; **D**. syntenic analysis, including orthologous genes, syntenic regions and triplicated blocks in *B. rapa* and *A. thaliana*; **E**. the orthologous genes of *Bol007288* in *A. thaliana* (*AT5G06860* and *AT3G12090*); **F**. the orthologous genes of *Bol007288* in *B. rapa* (*Bra038699* and *Bra000594*).

A total of 45,758 predicted genes with annotations were collected in Bolbase (Table 1). Putative genes with a variety of architectonic types, such as gene families, orthologous groups, and tandem arrays, and their locations on pseudo-molecular chromosomes and scaffolds were included in Bolbase. Each putative gene was annotated using public databases or web service sites to obtain a comprehensive functional overview (Figure 2). A total of 13,382 transposable elements in *B. oleracea* were deposited in Bolbase, including 2 major classes: retrotransposons (Class I transposons) and DNA transposons (Class II transposons). Additional categories, such as long terminal repeat retrotransposons (LTR-RTs), long interspersed nuclear elements (LINEs), short interspersed nuclear elements (SINEs), *Tc1-Mariner*, *hAT*, *Mutator*, *Pong*, *PIF-Harbinger*, CACTA, *Helitron*, and miniature inverted repeat transposable elements (MITEs) were hierarchically listed. Moreover, information on different superfamilies and families of LTR-RT elements was also provided. Bolbase compiled 3,581 non-coding RNAs by their conserved motifs and sequence similarities: 312 microRNAs (miRNAs), 517 ribosomal RNAs (rRNAs: 18S, 28S, 5.8S, and 5S), 1,434 small nuclear RNAs (snRNAs: CD-box, HACA-box, and splicing), and 1,318 transfer RNAs (tRNAs).

**Table 1:** Comparison of predicted protein-coding genes in *Brassica oleracea, Brassica rapa*, and *Arabidopsis thaliana*

**Species**	**Number of genes**	**Average transcript length (bp)**	**Average CDS length (bp)**	**Average exons per gene**	**Average exon length (bp)**	**Average intron length (bp)**
***B. oleracea***	45,758	1,761	1,037	4.55	228	204
***B. rapa***^**€**^	41,174	2,014	1,171	5.03	232	210
***A. thaliana***^**￡**^	27,379	2,176	1,215	5.38	237	235

^**€**^*B. rapa* genome V1.0 gene sets downloaded from BRAD (http://brassicadb.org/brad/).

^**￡**^*A. thaliana* genome TAIR9 representative gene sets downloaded from TAIR (http://www.arabidopsis.org/).

### Gene clusters

Clusters of genes with similar functions evolve through tandem, segmental, or whole genome duplication and are remarkably important for genome evolution and trait establishment. The gene cluster section in Bolbase is composed of gene families, orthologous groups, and tandem duplicated arrays. First, HMMER v3.0 software was employed to detect gene family members using HMM profile from the Pfam database [11,12]. Second, OrthoMCL 2.0 software was used to classify orthologous groups with E-value ≤ 1e-05 and inflation parameter of 1.5; all *B. oleracea* genes were divided into 21,509 ortholog groups [13]. Third, tandem duplicated genes were classified using the BLASTP program with E-value cutoff ≤ 1e-20 where one unrelated gene within a tandem array was allowed. Approximately 1,825 tandem arrays with 2 to 12 genes each were detected and saved in Bolbase.

### Syntenic regions

**Figure 3:**
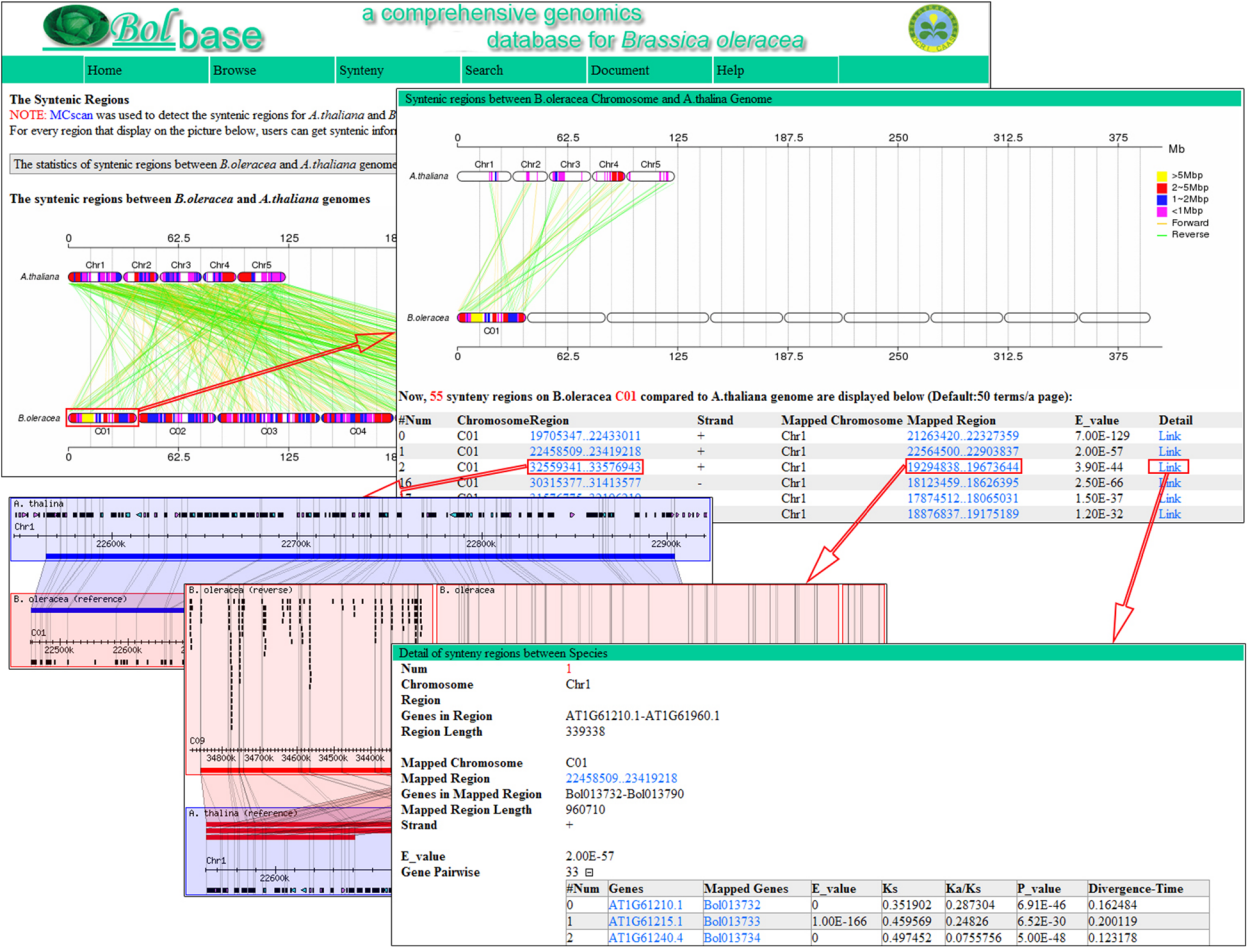
**Syntenic regions of *Brassica oleracea *chromosome C01 and the *Arabidopsis thaliana *genome.** As an example, *B. oleracea* chromosome C01, which contains 55 syntenic regions, was compared to the genome of *A. thaliana*. The hyperlinks under 'Region’ or 'Mapped Region’ will visually present the syntenic relationship between the two genomes*.* The hyperlinks under 'Detail’ will retrieve orthologous gene pairs in the syntenic regions and calculate their *Ka*/*Ks* values and divergence times.

To better understand evolutionary history and species divergence, syntenic regions between *A. thaliana* and *Brassica* species were identified using the MCscanX software and manual curation, and they can be visualized and used in Bolbase [14] (Figure 3). Orthologous gene pairs were first identified based on an all-against-all BLAST search with an E-value cutoff ≤ 1e-10 between species from best-reciprocal BLAST hits [15]. Then, MCscanX was employed to identify syntenic regions, using the parameters e = 1e-20, u = 1, and s = 5, which required a minimum of five consecutive orthologous gene pairs in the collinear regions. In total, 558 syntenic regions, including 22,413 gene pairs, were classified between *B. oleracea* and *A. thaliana*, and 1,034 syntenic regions containing 24,422 gene pairs were defined between *B. oleracea* and *B. rapa*. These data can be freely accessed and visualized (Table 2, Additional file 1). Moreover, nonsynonymous (*Ka*) and synonymous (*Ks*) substitution rates of orthologous gene pairs were calculated and provided.

**Table 2:** **Syntenic regions on pseudomolecular chromosomes in ***Brassica oleracea, Brassica rapa*, and ***Arabidopsis thaliana***

***B. oleracea***	***A. thaliana***					***B. rapa***									
**Chromosome ID**	**Chr1**	**Chr2**	**Chr3**	**Chr4**	**Chr5**	**A01**	**A02**	**A03**	**A04**	**A05**	**A06**	**A07**	**A08**	**A09**	**A10**
**C01**	6	10	12	15	12	20	3	18	7	11	14	4	16	7	7
**C02**	14	4	8	9	19	5	21	8	1	7	12	15	6	13	8
**C03**	12	13	26	25	16	25	12	28	10	23	17	6	12	17	15
**C04**	9	22	20	3	6	3	1	14	30	18	3	13	3	15	3
**C05**	28	5	9	2	4	9	4	10	4	20	14	7	10	13	5
**C06**	14	14	9	15	17	15	9	15	3	3	14	12	15	31	7
**C07**	35	4	16	4	1	3	9	6	9	14	21	30	13	11	2
**C08**	31	11	8	8	3	10	1	9	8	7	25	17	22	21	4
**C09**	4	4	9	13	29	10	24	21	–	4	9	3	1	16	13

'–’: no syntenic region between the corresponding chromosomes.

## Utility

Bolbase provides a user-friendly interface to facilitate the retrieval of information. Five main functional units —browse, synteny, search, document, and help — were integrated into Bolbase. From those units, users can browse genomic and comparative genomic information for *B. oleracea* and its relatives or retrieve comprehensive genomic component annotations, their locations on pseudomolecular chromosomes, and genome sequences. These genomic data can also be downloaded in bulk. Therefore, Bolbase will facilitate studies on genome variation and genomic structure differentiation within and between species. Here we describe some main functions of the interface.

### Browsing genomic components and syntenic regions

The genomic component web interface of Bolbase is organized by component type. Each of the main navigation tabs focuses on a specific component to allow users to retrieve information from the database. This functional unit is contained in "Browse" on the main navigation bar. The putative gene tab is organized by gene families, orthologous groups, tandem arrays, and gene locations on pseudomolecular chromosomes or scaffolds. Repeat element and non-coding RNA tabs are organized by types, categories, or superfamilies. IN particular, Bolbase provides detailed function annotations for every putative gene that can be divided into four units: (i) basic information (Figure 2A); (ii) protein sequence features (Figure 2B); (iii) gene clusters, including orthologous groups and tandem duplicated arrays (Figure 2C); and (iv) syntenic analyses including orthologs in *B. rapa* and *A. thaliana*, as well as corresponding syntenic regions and triplicated blocks (Figure 2D). Basic information consists of gene identifier, location, model structure (intron/exon boundary, number, length, etc.), and coding nucleotide and protein/peptide sequences. The unit of protein sequence features displays conserved protein domains or motifs predicted by InterProScan in detail [16]. Additionally, putative genes were also annotated and compared with different databases, including Gene Ontology (GO) [17], Swiss-Prot [18], TrEMBL [18] and Kyoto Encyclopedia of Genes and Genomes (KEGG) [19].

To better visualize the collinear relationship between species, the syntenic regions in *B. oleracea*, *B. rapa*, and *A. thaliana* are visualized on chromosomal images produced by Perl scripts, and statistical analyses of gene pairs between species are also scatter plotted. The syntenic regions between any target chromosome and those of other species will appear when the chromosome is selected, revealing gene pairs in each region and their *Ka*, *Ks* and *Ka*/*Ks* values.

### Keyword search

The keyword search is a powerful search engine to retrieve useful information, such as sequences, annotations, and homologous genes. These functional units are contained in the "Search" section on the main navigation bar. This section mainly includes putative gene, transposable element, and non-coding RNA search pages. Putative gene searching will provide users with detailed annotations, orthologous genes, and/or tandem arrays, if they exist. By inputting a GO term, a InterPro entry, or a KEGG pathway entry, researchers can retrieve a group of putative genes in the *B. oleracea* genome. Different types, categories, and superfamilies of transposable elements can be screened in the transposable element search page. The non-coding RNA search page is designed to help users compile information on these genetic elements. The different types or categories of non-coding RNA can be also searched on this page.

### Orthologous genes and syntenic regions search

Through comparative analyses among species, researchers can further understand the genomes of *B. oleracea* and its relatives. Orthologous genes in conserved syntenic regions can be displayed using a localized GBrowse_syn software by inputting a gene name, as indicated in Figure 3[20,21]. This functional unit is contained in the "Search" section on the main navigation bar. Here, we use the *B. oleracea* gene Bol007288 as an example to show orthologous genes in related species. By searching with Bol007288 as query on the orthologous genes search page, two orthologs in *A. thaliana* (AT5G06860 and AT3G12090) and two in *B. rapa* (Bra038699 and Bra000594) are retrieved (Figure 2E,F). By selecting a chromosome from one species, syntenic regions in the other species can be visualized as a comparative chromosomal image, and lists of syntenic regions are displayed with their chromosomal positions. When the hyperlink for the target region is clicked, the syntenic regions in other species will be displayed.

### Sequence similarity search

The similarity search page, which embeds customized BLAST software, will satisfy users with various interests related to homologous genes or regions. This functional unit is contained in the "Search" section on the main navigation bar. Users can supply a nucleic acid or amino acid sequence by uploading or directly pasting it to search against the available databases. Thus, this function allows quick comparisons and annotations of user query sequences using the data deposited in Bolbase. BLAST hits return with hyperlinks to the genes, enabling users to quickly acquire annotations from the database.

## Discussion

Although a few *Brassica* databases existed previously, Bolbase is the first comprehensive database with a focus on the *B. oleracea* genome and comparisons with its relatives. The deposited sequences and relatively accurate annotations will allow users to retrieve and download important information to further their interests in both functional and comparative genomics studies. Compared to other databases of *B. oleracea* genomic data, Bolbase supplies more detailed genomic annotations from public databases to allow users to analyze them more thoroughly. Syntenic regions and orthologous genes, which are useful resources for comparative and evolutionary analysis, can be explored in a highly visual style. Additionally, the user-friendly interface provides users quick and comprehensive information. The friendly and powerful search tools allow multi-channel searching and will be improved in the future based on user feedback. We continue to update and expand the database by adding data from other *Brassica* species as they become available.

## Conclusions

We have developed Bolbase, a comprehensive and searchable database of the *B. oleracea* genome. Bolbase is the primary resource platform for the *B. oleracea* genome and for genomic comparisons with its relatives, and its functions are not available in other public databases of *Brassica* species. To assist researchers and breeders in using the *B. oleracea* genomic information efficiently, Bolbase will be regularly updated with new genome annotations and the results of comparisons with newly-sequenced genomes as they become available. We hope that Bolbase will provide a valuable resource for the study of the functional and evolutionary aspects of *Brassica* genomes and for further exploration of the evolutionary relationships within the *Brassica* genus and the crucifer lineage.

## Availability and requirements

**Database:** Bolbase.

**Database homepage:**http://ocri-genomics.org/bolbase.

**Operating system(s):** Linux.

**Programming language:** Perl, Python, JavaScript.

**Other requirements:** Apache, PHP, MySQL, GD, SVG, GBrowse.

These data are freely available without restrictions for use by academics. Please login to the 'Help’ page on the Bolbase homepage or email Dr. Shengyi Liu (liusy@oilcrops.cn) to request data subsets of interest.

## Competing interests

The authors declare that they have no competing interests.

## Authors’ contributions

SL and JY conceived the study. JY collected the data, developed the database, JY and MZ prepared the manuscript. SL and WH revised the manuscript. XW, CT, SH, ST and YL prepared the basic datasets. All authors read and approved the final manuscript.

## Supplementary Material

Additional file 1 **Summary of syntenic regions in*Brassica oleracea*,*Brassica rapa,*and*Arabidopsis thaliana.*** In this Excel file, the "A.thaliana-B.oleracea_aligns" sheet is a summary of syntenic regions between the *B. oleracea* and *A. thaliana* genomes; the "B.oleracea-B.rapa_aligns" sheet is a summary of syntenic regions between the *B. oleracea* and *B. rapa* genomes; and the "A.thaliana-B.rapa_aligns" sheet is a summary of syntenic regions between the *B. rapa* and *A. thaliana* genomes. Click here for file

## References

[B1] U N Genome analysis in brassica with special reference to the experimental formation of B. Napus and peculiar mode of fertilization Japan J Bot 1935 7 389 452

[B2] Kalloo G Bergh BO Genetic improvement of vegetable crops 1993 Oxford: Pergamon

[B3] Wang XTM Pierce G Lemke C Nelson LK Yuksel B Bowers JE Marler B Xiao Y Lin L Epps E Sarazen H Rogers C Karunakaran S Ingles J Giattina E Mun JH Seol YJ Park BS Amasino RM Quiros CF Osborn TC Pires JC Town C Paterson AH A physical map of Brassica oleracea shows complexity of chromosomal changes following recursive paleopolyploidizations BMC Genomics 2011 12 470 485 10.1186/1471-2164-12-470 21955929 PMC3193055

[B4] Ayele MHB Kumar N Wu H Xiao Y Aken SV Utterback TR Wortman JR White OR Town CD Whole genome shotgun sequencing of Brassica oleracea and its application to gene discovery and annotation in Arabidopsis Genome Res 2005 15 487 495 10.1101/gr.3176505 15805490 PMC1074363

[B5] Bowers JE Chapman BA Rong J Paterson AH Unravelling angiosperm genome evolution by phylogenetic analysis of chromosomal duplication events Nature 2003 422 6930 433 438 10.1038/nature01521 12660784

[B6] Yau-Wen Yang K-NL Pon-Yean T Wen-Hsiung L Rates of nucleotide substitution in angiosperm mitochondrial DNA sequences and dates of divergence between brassica and other angiosperm lineages J Mol Evol 1999 48 597 604 10.1007/PL00006502 10198125

[B7] Tae-Jin Yang JSK Soo-Jin K Ki-Byung L Beom-Soon C Jin-A K Mina J Sequence-level analysis of the diploidization process in the triplicated FLOWERING LOCUS C region of brassica Rapa Plant Cell 2006 18 1339 1347 10.1105/tpc.105.040535 16632644 PMC1475497

[B8] Inaba RNT Phylogenetic analysis of brassiceae based on the nucleotide sequences of the S-locus related gene, SLR1 Theor Appl Genet 2002 105 8 1159 1165 10.1007/s00122-002-0968-3 12582894

[B9] Cheng F Liu S Wu J Fang L Sun S Liu B Li P Hua W Wang X BRAD, the genetics and genomics database for brassica plants BMC Plant Biol 2011 11 136 10.1186/1471-2229-11-136 21995777 PMC3213011

[B10] Huala E Dickerman AW Garcia-Hernandez M Weems D Reiser L LaFond F Hanley D Kiphart D Zhuang M Huang W The Arabidopsis information resource (TAIR): a comprehensive database and web-based information retrieval, analysis, and visualization system for a model plant Nucleic Acids Res 2001 29 1 102 105 10.1093/nar/29.1.102 11125061 PMC29827

[B11] Finn RD Clements J Eddy SR HMMER web server: interactive sequence similarity searching Nucleic Acids Res 2011 39 Web Server issue W29 37 21593126 10.1093/nar/gkr367 PMC3125773

[B12] Punta M Coggill PC Eberhardt RY Mistry J Tate J Boursnell C Pang N Forslund K Ceric G Clements J The Pfam protein families database Nucleic Acids Res 2012 40 Database issue D290 301 22127870 10.1093/nar/gkr1065 PMC3245129

[B13] Li L Stoeckert CJ Jr Roos DS OrthoMCL: identification of ortholog groups for eukaryotic genomes Genome Res 2003 13 9 2178 2189 10.1101/gr.1224503 12952885 PMC403725

[B14] Wang Y Tang H Debarry JD Tan X Li J Wang X Lee TH Jin H Marler B Guo H MCScanX: a toolkit for detection and evolutionary analysis of gene synteny and collinearity Nucleic Acids Res 2012 40 7 e49 10.1093/nar/gkr1293 22217600 PMC3326336

[B15] Altschul SF Madden TL Schaffer AA Zhang J Zhang Z Miller W Lipman DJ Gapped BLAST and PSI-BLAST: a new generation of protein database search programs Nucleic Acids Res 1997 25 17 3389 3402 10.1093/nar/25.17.3389 9254694 PMC146917

[B16] Quevillon E Silventoinen V Pillai S Harte N Mulder N Apweiler R Lopez R InterProScan: protein domains identifier Nucleic Acids Res 2005 33 Web Server issue W116 120 15980438 10.1093/nar/gki442 PMC1160203

[B17] Ashburner M Ball CA Blake JA Botstein D Butler H Cherry JM Davis AP Dolinski K Dwight SS Eppig JT Gene ontology: tool for the unification of biology. The gene ontology consortium Nat Genet 2000 25 1 25 29 10.1038/75556 10802651 PMC3037419

[B18] O’Donovan C Martin MJ Gattiker A Gasteiger E Bairoch A Apweiler R High-quality protein knowledge resource: SWISS-PROT and TrEMBL Brief Bioinform 2002 3 3 275 284 10.1093/bib/3.3.275 12230036

[B19] Kanehisa M Araki M Goto S Hattori M Hirakawa M Itoh M Katayama T Kawashima S Okuda S Tokimatsu T KEGG for linking genomes to life and the environment Nucleic Acids Res 2008 36 Database issue D480 484 18077471 10.1093/nar/gkm882 PMC2238879

[B20] Donlin MJ Using the generic genome browser (GBrowse) Curr Protoc Bioinformatics 2009 28 9.9.1 9.9.25 19957275 10.1002/0471250953.bi0909s28

[B21] McKay SJ Vergara IA Stajich JE Using the generic synteny browser (GBrowse_syn) Curr Protoc Bioinformatics 2010 31 9.12.1 9.12.25 10.1002/0471250953.bi0912s31 PMC3162311 20836076

